# Deep Functional Profiling Facilitates the Evaluation of the Antibacterial Potential of the Antibiotic Amicoumacin

**DOI:** 10.3390/antibiotics9040157

**Published:** 2020-04-02

**Authors:** Stanislav S. Terekhov, Anton S. Nazarov, Yuliana A. Mokrushina, Margarita N. Baranova, Nadezhda A. Potapova, Maja V. Malakhova, Elena N. Ilina, Ivan V. Smirnov, Alexander G. Gabibov

**Affiliations:** 1 Shemyakin-Ovchinnikov Institute of Bioorganic Chemistry of the Russian Academy of Sciences, Moscow 117997, Russia; nazarov.ngu@gmail.com (A.S.N.); yuliana256@mail.ru (Y.A.M.); Baranova@ibch.ru (M.N.B.); smirnov@ibch.ru (I.V.S.); 2 Department of Chemistry, Lomonosov Moscow State University, Moscow 119991, Russia; 3 Institute for Information Transmission Problems (Kharkevich Institute) of the Russian Academy of Sciences, Moscow 127051, Russia; nadezhdalpotapova@gmail.com; 4 Federal Research and Clinical Centre of Physical-Chemical Medicine of Federal Medical Biological Agency, Moscow 119435, Russia; maja_m@mail.ru (M.V.M.); ilinaen@gmail.com (E.N.I.); 5 Department of Life Sciences, Higher School of Economics, Moscow 101000, Russia

**Keywords:** deep functional profiling, ultrahigh-throughput screening, amicoumacin, antibiotic activity spectrum, amide stability toward hydrolysis, single-cell, multi-omics

## Abstract

The global spread of antibiotic resistance is forcing the scientific community to find new molecular strategies to counteract it. Deep functional profiling of microbiomes provides an alternative source for the discovery of novel antibiotic producers and probiotics. Recently, we implemented this ultrahigh-throughput screening approach for the isolation of *Bacillus pumilus* strains efficiently producing the ribosome-targeting antibiotic amicoumacin A (Ami). Proteomics and metabolomics revealed essential insight into the activation of Ami biosynthesis. Here, we applied omics to boost Ami biosynthesis, providing the optimized cultivation conditions for high-scale production of Ami. Ami displayed a pronounced activity against *Lactobacillales* and *Staphylococcaceae*, including methicillin-resistant *Staphylococcus aureus* (MRSA) strains, which was determined using both classical and massive single-cell microfluidic assays. However, the practical application of Ami is limited by its high cytotoxicity and particularly low stability. The former is associated with its self-lactonization, serving as an improvised intermediate state of Ami hydrolysis. This intramolecular reaction decreases Ami half-life at physiological conditions to less than 2 h, which is unprecedented for a terminal amide. While we speculate that the instability of Ami is essential for *Bacillus* ecology, we believe that its stable analogs represent attractive lead compounds both for antibiotic discovery and for anticancer drug development.

## 1. Introduction

The global spread of antibiotic resistance (AR) is one of the most urgent problems faced by humanity, particularly relevant to the so-called ESKAPE pathogens (*Enterococcus faecium*, *Staphylococcus aureus*, *Klebsiella pneumoniae*, *Acinetobacter baumannii*, *Pseudomonas aeruginosa*, and *Enterobacter* species). Globally, antibiotic-resistant infections cause 31.5 million sepsis cases resulting in 5.3 million deaths annually [1]. Hence, the World Health Assembly endorsed a global action plan to tackle AR [2]. The efficient counteraction of the AR problem demands the creation of new molecular tools with antibacterial activity. While classical platforms for antibiotic discovery have been successfully applied, providing the majority of antibiotics on the market [3], a new paradigm of studying microbiome function at the single-cell level is required [4]. Recently, we proposed a concept of deep functional microbiome profiling based on single-cell cultivation of microorganisms encapsulated in isolated compartments of a double water-in-oil-in-water emulsion, combined with subsequent probing of their functionality [5]. Previously, ultrahigh-throughput profiling of bacterial communities for anti-*Staphylococcus* activity enabled isolating particular clones with the highest level of antibiotic production [6]. The application of this technology to the screening of the exotic microbiome of the East Siberian brown bear (*Ursus arctos collaris*) resulted in the isolation of *Bacillus pumilus* strains efficiently producing the antibiotic amicoumacin A (Ami) [5]. While Ami was discovered in the 1980s [7,8,9], its mechanism of action and molecular target remained ambiguous for a long time [10] and were precisely determined just recently [11]. Ami efficiently targets both pro- and eukaryotic ribosomes [11,12], resulting in similar minimum inhibitory concentrations (MICs) and IC_50_ values for Ami-sensitive bacterial strains and human cell lines, respectively [12]. This cytotoxicity restricts its application as an antibiotic on the one hand and provides a perspective agent targeting mammalian translation on the other. However, Ami was reported as an effective anti-inflammatory and antiulcer agent [7], which was subsequently associated with its anti-*Helicobacter pylori* activity [13]. Numerous *Bacillus* strains produce Ami [14] and are applied as probiotics to enhance gastrointestinal health [13] or to prevent *Vibrio* infections in aquaculture [15,16]. Whereas the immunostimulatory effects of *Bacillus* resulting in an increase in IgA level were reported [17], the antibiotic activity of Ami is a more straightforward and documented reason for the efficacy of Ami-producing *Bacillus* probiotics [15,16].

The origin of Ami biosynthesis has been of interest for a long time [14]. However, Ami biosynthetic gene cluster was firstly identified in *Bacillus subtilis* not so long ago [18]. Recently, we described a related Ami cluster in *B. pumilus*, clarifying the mechanisms mediating Ami biosynthesis in *Bacilli* [5]. A multi-omics approach applied to discover the regulation of Ami production revealed an increased level of the putative AmiP transporter, activating the peptidase AmiB [18,19,20], and a reversed balance between the kinase AmiN and the phosphatase AmiO upon activation of Ami biosynthesis [5]. Finally, we showed that AmiN (EC 2.7.1.230) and AmiO (EC 3.1.3.107) are responsible for self-resistance and corresponding Ami activation.

In this paper, we focused on the optimization of *B. pumilus* cultivation conditions for improved high-scale production of Ami based on the proteomics and metabolomics data obtained previously [5]. A combination of increased aeration, high-carbohydrate medium, and calcium carbonate microparticles mimicking the thin-layer cultivation previously described was efficiently applied to activate Ami biosynthesis and boost its production. The optimized cultivation conditions were implemented for high-scale preparation of Ami, simplifying the downstream purification based on reversed-phase solid-phase extraction (SPE) and HPLC. The purified Ami was used for the evaluation of its antibacterial potential using standard cultivation assays and AR-based deep functional profiling of microbiomes. Ami activity spectrum was studied previously [7,10,13,15], indicating potent activity of Ami against *H. pylori*, *Vibrio*, and *S. aureus* strains, including MRSA. We expand this data illustrating that Ami is active against numerous Gram-positive bacteria, including the overwhelming majority of pathogenic *Enterococcus* and *Staphylococcus* strains. However, we suggest that the application of Ami as an antibiotic or anticancer agent is problematic, since it has low stability in aqueous solutions at physiological pH. We observed the sequential transformation of Ami into AmiC and AmiB, which are inactive metabolites of Ami (lactone and carboxylic acid, respectively). The outstanding instability of the terminal amide of Ami originates from its intramolecular self-lactonization. This process is exceptionally efficient in the case of Ami, decreasing its half-life to less than 2 h at physiological conditions. We speculate that this self-lactonization is so productive because of the formation of the energetically favorable five-membered transition state. Moreover, the proximate protonated amine group enhances this effect. Hence, we consider that self-lactonization serves as an improvised intermediate state, substantially decreasing the energetic barrier of amide hydrolysis. While the exact mechanism of this reaction should be detailed in the future, a balance between antibiotic potency and stability seems to be necessary for Ami-producing *Bacillus*. However, it represents a great obstacle for the practical application of Ami as an antibiotic and anticancer drug.

## 2. Results

### 2.1. High-Scale Ami Production and Purification

**Figure 1:**
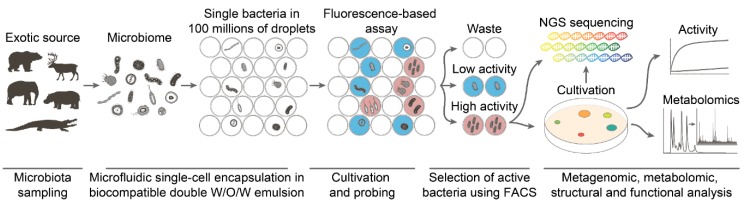
Pipeline of deep functional microbiome profiling. An exotic microbiota undergoes single-cell encapsulation in droplets of a biocompatible microfluidic double emulsion with the respective fluorogenic reporters. A fluorescence-based assay enables probing of the biological or biochemical activity of the encapsulated microorganisms. FACS-based screening classifies the microbiome based on the phenotype assayed. The selected subpopulations are analyzed subsequently by a combination of omics technologies for a detailed characterization of their phenotypes. NGS: Next Generation Sequencing.

The deep functional microbiome profiling method reported recently [5] consists of encapsulating single microorganisms of exotic microbiomes in isolated biocompatible microcompartments, estimating their biological or biochemical activity with fluorescent probes, and selecting subpopulations of interest based on a combination of fluorescent reporters (Figure 1). This technology is based on the application of droplet microfluidics allowing the generation of double water-in-oil-in-water (W/OW) emulsion droplets with precisely controlled size, in particularly mild emulsification conditions. Droplets serve as microbioreactors in which cultivation, co-cultivation, biocatalytic assays, or vital staining can be carried out. Specific combinations of fluorescent reporters indicate the functionality of the encapsulated species. The combinations of fluorescent reporters trigger FACS-assisted selection of the populations of interest. The selected populations are analyzed by classical cultivation-based assays including downstream activity profiling, proteomics, and metabolomics or examined by whole-genome sequencing and bioinformatics to trace unculturable or slow-growing species.

**Figure 2:**
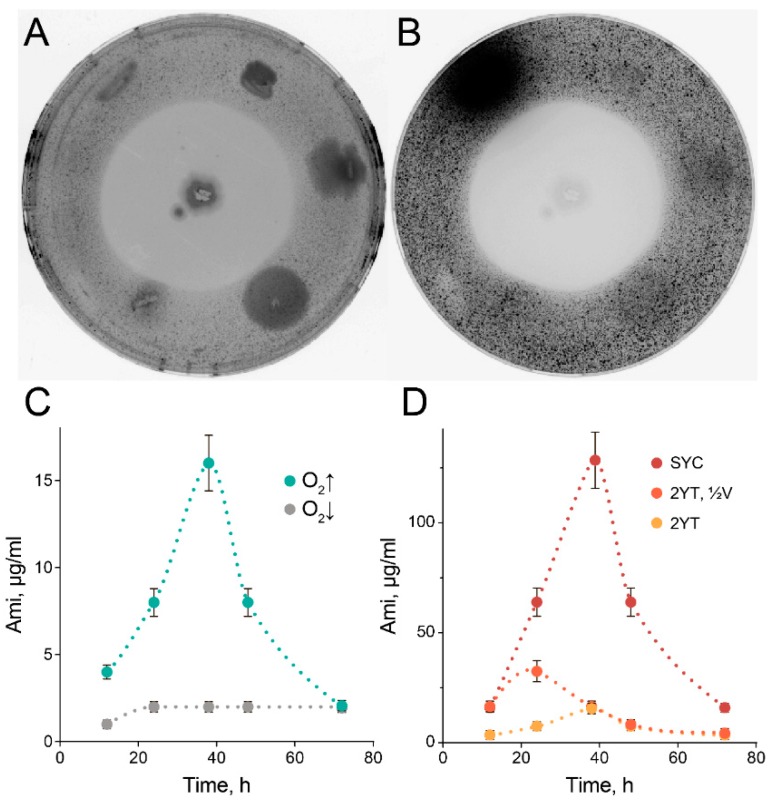
The selected *Bacillus pumilus* efficiently produce Ami. Cultivation of Ami-producing *B. pumilus* on agar causes the appearance of substantial zones of clearance. The clearance zones were observed with the agar overlay assay using a reporter *Staphylococcus aureus* strain producing GFP in visible light (**A**) and by fluorescence analysis of GFP (**B**). The colonies around *B. pumilus* (center) are the representative strains picked from the oral microbiome of brown bear, randomly. The data illustrate the representative view of three independent repeats. (**C**) Cultivation of *B. pumilus* in limited (grey) or high (aquamarine) aeration conditions. (**D**) Cultivation of *B. pumilus* at high aeration in SYC medium (red) and 2YT medium in the same volume (yellow) or in a twice reduced volume (orange). Ami concentration (dots) was estimated by an antibacterial activity assay of culture medium in triplicate. Data represent mean ± SD.

The described functional profiling platform was applied for the isolation of Ami-producing *B. pumilus* strains, and we exploited it to select the most efficient Ami producers from the same source of the microbiota. We isolated more than 50 Ami-producing *B. pumilus* clones totally, which were evaluated for Ami production. About 28% of them had a similar level of antagonistic activity, and we associated them with the same strain of origin. The isolated *B. pumilus* strains produced remarkable (>40 mm) zones of clearance that were observed using a reporter *S. aureus* (MRSA) strain (Figure 2A, 2B). However, cultivation in a nutritionally rich 2YT medium with limited aeration resulted in mediocre Ami production (Figure 2C), while increased aeration facilitated Ami production by more than sixfold (Figure 2C). Hence, we concluded that the cultivation conditions are particularly important for efficient Ami production. Previous proteomics observations [5] indicated that the activation of inosine monophosphate (IMP) biosynthesis, peptidoglycan synthesis, as well as flagellum organization correlate with cultivation in the thin layer associated with Ami production. Taking this into account, we suggested boosting the activation of Ami biosynthesis by high carbohydrate concentrations, in turn mediating the activation of IMP synthesis and facilitating the formation of peptidoglycan [21]. Previously, we associated flagellum organization in *B. pumilus* with biofilm formation. Hence, we reasoned that stimulation of cell–cell contacts is essential for high-scale Ami production, and this results in differences in Ami production during cultivation in liquid and semi-liquid growth medium. We stimulated cell–cell interactions and adhesion by the addition of calcium carbonate microparticles. The resulting SYC medium allowed a more than eightfold increase in Ami production in comparison with 2YT medium (Figure 2D). The concentration of Ami in 2YT medium could be increased by cultivation in shaking flasks in a decreased volume. However, this will not be convenient for Ami high-scale production.

**Figure 3:**
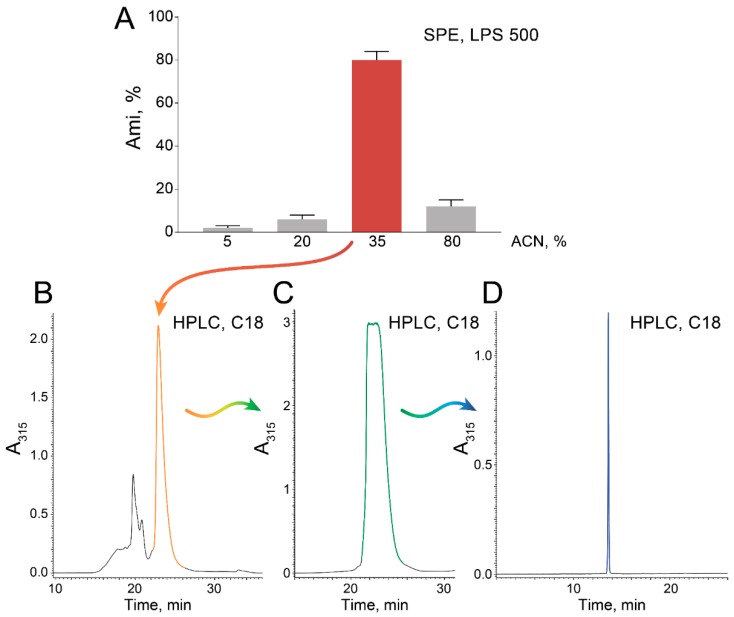
Optimized procedure of Ami purification. (**A**) Solid-phase extraction (SPE) with the polystyrene-based resin LPS 500 (Technosorbent, Russia). The portion of Ami eluted by the corresponding gradient step is indicated. (**B**,**C**) Sequential purification of Ami-containing fraction using a C18 RP-HPLC column. (**D**) Chromatogram of the purified Ami sample. Ami yield was estimated by an antibacterial activity assay in triplicate. Data represent mean ± SD.

The subsequent purification was simplified by solid-phase extraction (SPE) with a polystyrene-based resin (Figure 3A) followed by subsequent polishing on a C18 HPLC column (Figure 3B–D).

SPE resulted in a highly Ami-enriched fraction eluted with 35% acetonitrile (ACN), which was efficiently purified by C18 RP-HPLC, resulting in pure Ami (Figure 3D). These steps are scalable and could be adopted for extensive bioproduction.

### 2.2. The Activity Spectrum of Ami

**Figure 4:**
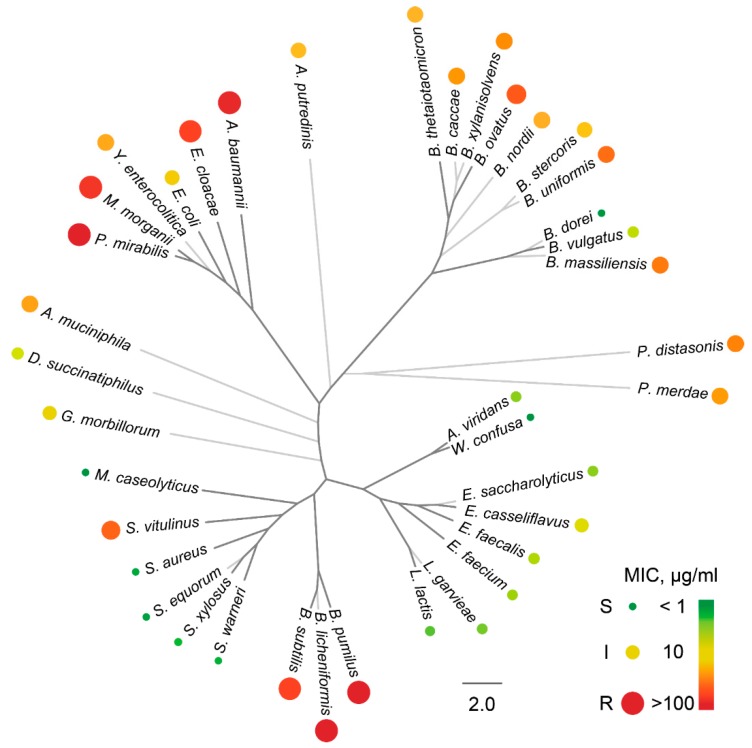
Comprehensive overview of Ami activity spectrum. The phylogeny of bacteria is presented with the respective mean minimum inhibitory concentrations (MICs). The color and area of circle markers indicate the average MIC value. Sensitive, intermediate, and resistant bacteria are indicated according to the color bar and marked as S, I, and R, respectively. The phylogenetic tree was reconstructed based on 16S rRNA. Bacteria analyzed solely using a bioinformatics-based deep functional profiling approach and not confirmed by standard cultivation assays are colored in light gray.

The purified Ami was used to characterize its activity spectrum using standard cultivation assays and deep functional profiling of microbiomes for Ami resistance. Deep functional profiling of microbiomes for Ami resistance is based on the cultivation of single bacteria from a particular microbiome in droplets with various concentrations of Ami, followed by their viability staining, FACS-based selection, metagenomic sequencing, and quantitative estimation of live bacteria via bioinformatics [5]. Here, we used a broad panel of clinical isolates to provide a more comprehensive description of the Ami activity spectrum (Figure 4).

Generally, Ami is active against Gram-positive non-spore-forming bacteria, including *Lactobacillales* and *Staphylococcaceae*. Ami is inactive toward *Bacillus* that we associate with homologs of AmiN kinase reported previously. Ami displayed antibacterial activity against some laboratory strains of *Escherichia coli* [11]. However, it showed only mediocre activity against *E. coli* wild strains. Moreover, Ami was ineffective against an especially valuable Gram-negative cohort of ESKAPE pathogens (*K. pneumoniae*, *A. baumannii*, *P. aeruginosa*, and *Enterobacter* species), while it was highly active against the overwhelming majority of *Enterococcus* and *Staphylococcus* strains (referred to as “E” and “S” ESKAPE pathogens, respectively). These bacteria represent the most clinically relevant fraction of Gram-positive pathogens, particularly associated with AR. Distinct *Bacteroides*, including *Bacteroides dorei* and *Bacteroides vulgatus*, were also sensitive to Ami (Figure 4). However, the majority of strains representing mutualistic gastrointestinal microbiota components were resistant to Ami, indicating Ami limited potential against this critical microbiota component.

### 2.3. Exceptional Instability of Ami

**Figure 5:**
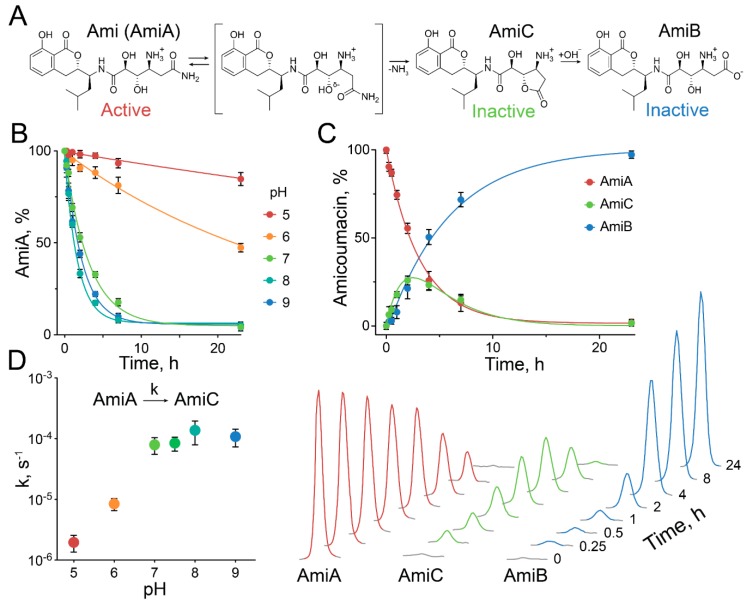
Ami is a particularly unstable amide, decomposing via intramolecular lactonization in physiological conditions. (**A**) General scheme, describing the sequential transformation of biologically active Ami (AmiA) into inactive amicoumacins AmiC and AmiB, respectively. (**B**) pH dependence of Ami decomposition. The residual portion of Ami over time is presented. Color indicates pH. (**C**) Representative time course of the conversion of AmiA into AmiC and AmiB, taking place at pH 7.5. The corresponding percentages of AmiA, AmiC, and AmiB in the reaction mixtures are presented. Amicoumacins were analyzed by HPLC (dots). The data were approximated by a two-step sequential reaction model (lines). The concentrations of amicoumacins were analyzed in triplicate. Data represent mean ± SD. (**D**) pH dependence of the rate-limiting step of Ami decomposition. The kinetic constants of the self-conversion of AmiA into AmiC are presented for pH 5–9. Data represent mean ± SD.

It was previously reported [9,20], and we also observed [5], that Ami (AmiA) is not stable in aqueous solutions and transforms into biologically inactive products, i.e., amicoumacin B (AmiB) and amicoumacin C (AmiC) (Figure 5A).

Ami has a terminal amide bond that is exceptionally unstable, and we decided to study this feature in detail. We observed that Ami decomposition is pH-dependent, accelerating rapidly from low acidic to neutral pH and reaching a maximum at pH 7–9 (Figure 5B,D). The analysis of Ami decomposition kinetics revealed that Ami spontaneously converts into lactone AmiC, which subsequently hydrolyzes to AmiB (Figure 5C). Our data indicate that even if direct conversion of Ami into AmiB was observed, it should be more than an order of magnitude slower than the transition of Ami into AmiC. Hence, the rate-limiting step of Ami hydrolysis is the first reaction, resulting in AmiC formation by intramolecular lactonization. We suggest that Ami structure determines Ami instability by the formation of the energetically favored five-membered ring transition state, accelerating its self-inactivation by lactonization (Figure 5A). Hence, AmiC lactone acts as an improvised intermediate state, substantially decreasing the energetic barrier of amide hydrolysis. We suppose this self-lactonization step provides the main contribution to Ami instability, decreasing its half-life from years, as reported for other terminal amides [22], to less than 2 h at pH 8 and 37 °C. The observed pH dependence indicates the potential impact of the Ami amine group on self-lactonization. This amine group has an estimated pKa of ~7.2. We suggest its protonation is critical for the self-conversion of Ami into AmiC, accelerating when pH increases from 5 to 7 and reaching a maximum at pH 7–9. We speculate that the effect of the protonated amine is mediated by intramolecular stabilization of the –OH^δ-^ nucleophile. Alternatively, the protonated amine could serve as an intramolecular proton donor for the ammonia leaving group. However, the detailed molecular mechanism of this particular Ami instability should be thoroughly investigated in the future and supported by quantum mechanics simulations and calculation of energetic profiles of Ami self-lactonization.

## 3. Discussion

Since its discovery at the beginning of the 1980s [7,8,9], Ami raised only limited practical interest, predominantly in academia. Recently, the ribosome-targeting mechanism of Ami was elucidated both for pro- [11] and for eukaryotes [12]. Furthermore, the molecular mechanisms of Ami activation/attenuation [20] and self-resistance [5] were identified. Hence, we suggest that these recent advances provide new applications of Ami in the fields of synthetic biology, biotechnology, and biomedicine. The high-level production of Ami is of interest in these cases, and our results indicate how it could be achieved by the application of state-of-the-art microbiological technologies. Deep functional profiling facilitates the isolation of potent Ami producers, while proteomics and metabolomics provide an essential basis for boosting Ami production.

Ami is active against both bacteria and mammalian cells, and this is the fundamental problem limiting its broad application. Taking into account the antibiotic potential of Ami, we classify it as a potent agent against Gram-positive pathogenic strains of *Enterococcus* and *Staphylococcus*, including MRSA. Ami was also reported as an efficient anti-*H. pylori* antibiotic, providing attractive gastroprotective action [13]. However, the major obstacle to the practical application of Ami is its low stability at physiological conditions. This is also relevant to *H. pylori*, which neutralizes the acidic conditions of the stomach via urease activity. Ami has a half-life of ~2 h in neutral and slightly alkaline conditions. That is more than 10^4^ times lower than the respective values obtained for terminally amidated peptides [23]. Hence, the rate of Ami hydrolysis at physiological conditions is similar to that of spontaneous amide hydrolysis at 170 °С [24] and close to the hydrolysis rate achieved by artificial enzymes, i.e., proteolytic antibodies [23,25].

We suggest that the low stability of the terminal amide bond in Ami is an exclusive feature originating from its unique structure. The decomposition of Ami is a two-step process, including its self-lactonization, followed by lactone hydrolysis. The self-lactonization of Ami is the rate-limiting step that makes the main contribution to the acceleration of Ami hydrolysis. This is achieved by the formation of the lactone AmiC, simulating the transition state and reducing the energetic barrier of amide hydrolysis. We consider that the potential of Ami has been underestimated for a long time because of the decreased stability of Ami in biological fluids. While the instability of Ami is a stumbling block for its clinical application, we suggest this feature is not accidental. We speculate that Ami serves as an efficient biocontrol agent providing the benefit for *Bacillus* over its competitors in the wild. The instability of Ami is advantageous in this case, as it enables a precisely time-resolved control over its inhibitory activity. The total synthesis of natural products of the amicoumacin family was reported recently [26,27]. Hence, total organic synthesis could provide a vital source of new Ami analogs tailored for antimicrobial or anticancer drug discovery, reviving the interest in this antibiotic.

## 4. Materials and Methods

### 4.1. Microbiota Collection and Storage

All human donors provided written informed consent and were examined before collecting the fecal sample. The examination included: general blood test, biochemical blood test, ELISA blood screening for *Lamblias*, *Toksokara*, *Opisthorchis*, *Ascaris*, and *Trichinella*, hepatitis B and C test, HIV test, syphilis test, urinalysis, and stool screening for *Clostridium difficile*, *Campylobacter*, *Salmonella*, enteroinvasive *E. coli* (EIEC), *Shigella*, Rotavirus, Norovirus, Adenovirus, *Cryptosporidium*, *Cyclospora*, *Giardia*, and *Isospora*. The results were negative for the pathogens tested. Stool samples were suspended in sterile medium for microbiota cryopreservation, composed of Brain Heart Infusion (BHI) medium (BD, USA), 20% glycerol, and 30% heat-inactivated fetal bovine serum (FBS) (Gibco, USA). The oral microbiota of East Siberian brown bear (*Ursus arctos collaris*) was collected previously [5] and stored in liquid nitrogen.

### 4.2. Deep Functional Profiling

The selection of bacteria displaying anti-*S. aureus* activity was described in detail previously [6]. Briefly, target *S. aureus* cells producing a GFP reporter were vitally stained with sulfo-Cyanine5 NHS (Lumiprobe, Russia), washed, filtered using 20 μm solvent filters (A-313, IDEX, USA), and co-encapsulated with a microbiota suspension in droplets of microfluidic double emulsion (MDE), using 20 μm microfluidic chips produced via soft lithography. Microbiota samples were unfrozen directly before encapsulation, resuspended in BHI broth (BD, USA), and filtered through 40 μm cell strainers (Greiner Bio-One, USA). After overnight incubation at 35 °C, Calcein Violet AM (Thermo Fisher Scientific, Waltham, MA, USA) was added to the droplet emulsion to the final concentration of 10 μM. Subsequently, the droplets with simultaneous sCy5^high^, GFP^low^, and Calcein Violet^high^ fluorescence were sorted using a FACSAria III cell sorter (BD, USA). Bacterial colonies were regenerated after plating on BHI–agar (BD, USA) and tested for anti-*S. aureus* activity using the agar overlay assay. Bacterial clones of *B. pumilus* demonstrating inhibition of *S. aureus* growth were identified by mass spectrometry. The procedure of deep profiling of Ami activity was described in detail previously [5]. Briefly, microbiota samples were co-encapsulated with Ami inside the MDE droplets, followed by cultivation, Calcein Violet AM staining, and FACSAria III sorting. The collected droplets were frozen in liquid nitrogen, freeze-dried, and analyzed by whole-genome sequencing and bioinformatics, resulting in the quantification of bacteria in the samples. The abundance of bacteria in the samples was used for the estimation of MIC, as it was described previously [5].

### 4.3. NGS Sequencing and Bioinformatics

The selected MDE droplets were freeze-dried, and total DNA was isolated using the QIAamp DNA Investigator Kit (Qiagen, USA). Whole-genome amplification was performed using the REPLI-g Single Cell Kit (Qiagen, USA). Fragment libraries were prepared using the NEBNext^®^ DNA Library Prep Reagent Set for Illumina and the NEBNext^®^ Multiplex Oligos for Illumina^®^ (96 Index Primers) (Illumina, San Diego, CA, USA), according to the manufacturer’s instructions. The sequencing of libraries was performed using the genetic analyzer HiSeq2500, the HiSeq PE Cluster Kit v4 cBot and the HiSeq SBS Kit v4 (250 cycles) (Illumina, San Diego, CA, USA), according to the manufacturer’s instructions. Genome assemblies were performed using SPAdes 3.9.0 [28]. Bacteria abundance in metagenome samples was assessed with Metaphlan2 [29]. The bacterial phylogeny was reconstructed based on 16S rRNA. We used Muscle [30] for sequence alignment. Quality control of alignment was done in UGENE [31]. RaxML was used for phylogenetic tree reconstruction [32]. FigTree was used for phylogenetic tree visualization.

### 4.4. Identification of Bacteria using Mass Spectrometry

Bacterial cells were spotted on a sample spot of a MALDI target plate (MSP 96 target, ground steel; Bruker Daltonics, Billerica, MA, USA) and were overlaid with HCCA matrix solution (saturated solution of α-4-cyano-hydroxycinnamic acid; Bruker Daltonics, Billerica, MA, USA) in 50% acetonitrile (Sigma-Aldrich, St. Louis, MO, USA) and 2.5% trifluoroacetic acid solution (Sigma-Aldrich, St. Louis, MO, USA). Mass spectra profiles were acquired using a Microflex spectrometer (Bruker Daltonics, Billerica, MA, USA). The molecular ions were measured automatically in linear positive ion mode with the instrument parameters optimized for a range of 2,000–20,000 *m*/*z*. The software packages flexControl 3.0 (Bruker Daltonics, Billerica, MA, USA) and flexAnalysis 3.0 (Bruker Daltonics, Billerica, MA, USA) were used for mass spectra recording and processing. Spectra identification and analysis were carried out using the MALDI Biotyper 3.0 (Bruker Daltonics, Billerica, MA, USA). The identification was performed by comparing the obtained spectra with those in the MALDI Biotyper 3.0 library (version 3.2.1.1).

### 4.5. Antimicrobial Activity

A bacterial collection of clinical isolates was kindly provided by Lytech Co. Ltd. The MICs for the bacteria were determined by serial two-fold dilution (0.1–256 µg/mL) in Mueller–Hinton Broth (BD, USA) or Anaerobe Basal Broth (Oxoid, USA) in anaerobic conditions for *Bacteroides*. MIC was defined as the lowest concentration of amicoumacins that prevented the growth of the test organism in a 96-well plate after 10 h of cultivation. Prolonged cultivation is undesirable due to spontaneous Ami self-hydrolysis reaction and leads to overestimated MICs. The bacterial growth time course was monitored at 800 nm, using a Varioskan Flash multimode reader (Thermo Fisher Scientific, Waltham, MA, USA).

### 4.6. Ami Production and Purification

Ami producing *B. pumilus* was cultivated in 2YT medium (16 g/L tryptone, 10 g/L yeast extract, 5 g/L NaCl) or SYC medium containing 40 g/L sucrose, 5 g/L yeast extract, 4 g/L CaCO_3_, 1.5 g/L K_2_HPO_4_, 2 g/L glucose, 2 g/L NaCl, 1.5 g/L MgSO_4_, 2 g/L (NH_4_)_2_SO_4_, 0.01 g/L FeSO_4_, 0.01 g/L MnCl_2_, at 28 °C. *B. pumilus* was inoculated from an overnight culture (using 1:100 dilution) and cultivated using 750 mL flasks in 100 mL with 250 rpm shaking. The cells were centrifuged at 10,000× *g* for 10 min, and the supernatant was filtered using a Millistak + HC Pod Depth Filter (Millipore, Billerica, MA, USA). Ami purification included three steps of chromatography. In the first step, the supernatant was purified by SPE with LPS-500 sorbent (Technosorbent, Russia) on column XK 26 (GE Healthcare Life Sciences, Pittsburgh, PA, USA), using buffer A (10 mM NH_4_OAc pH 5.0, 5% ACN), buffer B (10 mM NH_4_OAc pH 5.0, 80% ACN), flow rate 6 mL/min, step gradient 0–10 min (0% B), 10–20 min (20% B), 20–36 min (40% B), and 36–45 min (100% B). The fractions containing Ami (40% B) were freeze-dried, dissolved in DMSO, and fractionated twice on an RP-HPLC Zorbax ODS 62 × 250 mm (Agilent, Santa Clara, CA, USA) column using buffer A and B, flow rate 5 mL/min, gradient 0–10 min (0% B), 10–24 min (0%–70% B), 24–25 min (70%–100% B). Finally, Ami was purified on a Symmetry C18 5 μm 4.6 × 150 mm (Waters) RP-HPLC column, using buffer A and B, flow rate 1 mL/min, gradient 0–5 min (0% B), 5–20 min (0%–100% B). Amicoumacin and its derivatives were monitored by absorbance at 315 nm. The concentration of Ami was measured using $\varepsilon_{315\mathrm{nm}}^{\mathrm{MeOH}}$ = 4380 M^−1^cm^−1^.

### 4.7. Ami Hydrolysis

Ami hydrolysis was performed at 37 °C in 20 mM buffers: NaOAc pH 5.0, NaOAc pH 6.0, Na-phosphate pH 7.0, Na-phosphate pH 7.5, Na-phosphate pH 8.0, Bis-Tris-Propane pH 9.0. The reaction mix was analyzed by RP-HPLC using the Symmetry C18 (Waters, USA) column (1 mL/min, linear gradient 5%–53% ACN with 20 mM NH_4_OAc pH 6.0, in 10 min). Amicoumacin and its derivatives were monitored by absorbance, assuming $\varepsilon_{315\mathrm{nm}}^{\mathrm{MeOH}}$ = 4380 M^−1^ cm^−1^. The retention times were as follows: AmiB –8.47 ± 0.03 min, Ami (AmiA)–9.00 ± 0.03 min, AmiC–9.90 ± 0.03 min.
